# Ionic-Liquid Gating in Two-Dimensional TMDs: The Operation Principles and Spectroscopic Capabilities

**DOI:** 10.3390/mi12121576

**Published:** 2021-12-17

**Authors:** Daniel Vaquero, Vito Clericò, Juan Salvador-Sánchez, Jorge Quereda, Enrique Diez, Ana M. Pérez-Muñoz

**Affiliations:** 1 Nanotechnology Group, USAL–Nanolab, Universidad de Salamanca, E-37008 Salamanca, Spain; danivaqu@usal.es (D.V.); vito_clerico@usal.es (V.C.); juan2s@usal.es (J.S.-S.); J.Quereda@usal.es (J.Q.); 2 FIW Consulting S.L., Gabriel Garcia Marquez, 4 las Rozas, E-28232 Madrid, Spain

**Keywords:** ionic liquid gating, ionic gate spectroscopy, ambipolar FETs, transition metal dichalcogenides

## Abstract

Ionic-liquid gating (ILG) is able to enhance carrier densities well above the achievable values in traditional field-effect transistors (FETs), revealing it to be a promising technique for exploring the electronic phases of materials in extreme doping regimes. Due to their chemical stability, transition metal dichalcogenides (TMDs) are ideal candidates to produce ionic-liquid-gated FETs. Furthermore, as recently discovered, ILG can be used to obtain the band gap of two-dimensional semiconductors directly from the simple transfer characteristics. In this work, we present an overview of the operation principles of ionic liquid gating in TMD-based transistors, establishing the importance of the reference voltage to obtain hysteresis-free transfer characteristics, and hence, precisely determine the band gap. We produced ILG-based bilayer WSe_2_ FETs and demonstrated their ambipolar behavior. We estimated the band gap directly from the transfer characteristics, demonstrating the potential of ILG as a spectroscopy technique.

## 1. Introduction

The discovery of two-dimensional materials unleashed a revolution in nanoelectronics during the last decade [1]. This family of materials holds enormous promise for the development of a new generation of semiconductor devices and, over the last few years, a considerable amount of effort has been invested in studying them and developing suitable devices that take advantage of their properties.

In 2011, Kis et al. demonstrated for the first time a field-effect transistor (FET) in which a bilayer MoS_2_ crystal was used as the semiconductor channel [2]. Since then, similar devices have been developed using several different two dimensional (2D) materials, and the device geometry, materials, and fabrication methods have been greatly improved [3].

However, FETs have certain fundamental limitations that cannot be easily overcome: the dielectric breakdown of the insulating layer and the presence of charged impurities between the gate electrode and the 2D channel results in a limited gating capability, which is often not sufficient to reach ambipolar response in 2D semiconductor devices. The technique of ionic-liquid gating (ILG) aims to overcome these fundamental limitations by replacing the dielectric material in conventional FETs with ionic liquids [4] with movable charged ions [5,6,7,8]. In recent years, ILG-based 2D transistors have been tested by a number of research groups, allowing them to achieve extremely large accumulations of charge carriers, up to 5 × 10^14^ electrons/cm^2^ while operating at moderate voltages within ±3 V [9].

Ionic-gating experiments have been widely used to control and investigate the electronic properties of oxides [7,10], nitrides [11], organic semiconductors [12,13,14], carbon-related materials [15], and III–V semiconductor nanowires [16,17,18,19]. The extreme tunability of charge carrier concentrations that can be obtained by this technique has allowed the attainment of new physical regimes, achieving, for example, superconductivity in band-insulating materials such as SrTiO_3_ (STO) [20], ZrNCl [11], or KTaO_3_ [21]. Currently, ILG has been established as a promising technique not only from an applied point of view, but also to obtain fundamental knowledge about the phase diagrams of novel materials [9,22]. More recently, ionic-gating experiments have moved forward through other inorganic systems, such as two-dimensional transition metal dichalcogenides (TMDs).

In this work, we present the operation principles for the use of ILG in TMD-based transistors. Due to their chemical stability, two-dimensional TMDs are ideal candidates to produce ionic liquid-gated FETs [23,24]. The very large geometrical capacitance of ionic liquid-gated devices allowed the observation of superconductivity in MoS2 [25,26,27,28,29,30,31] and WS2 [32,33,34], among other TMDs [35,36,37]. This technique has also enabled light emission by TMD-FETs operating in the ambipolar injection regime [38,39] and the enhancement of the electron−phonon interaction in multivalley TMDs [24,29,40]. Furthermore, as we present in this work, ILG-based TMD transistors grant the possibility of determining the band gap of semiconducting TMDs quantitatively from simple transport measurements [39,41,42,43,44].

## 2. Results

### 2.1. Device Fabrication and Geometry

**Figure 1:**
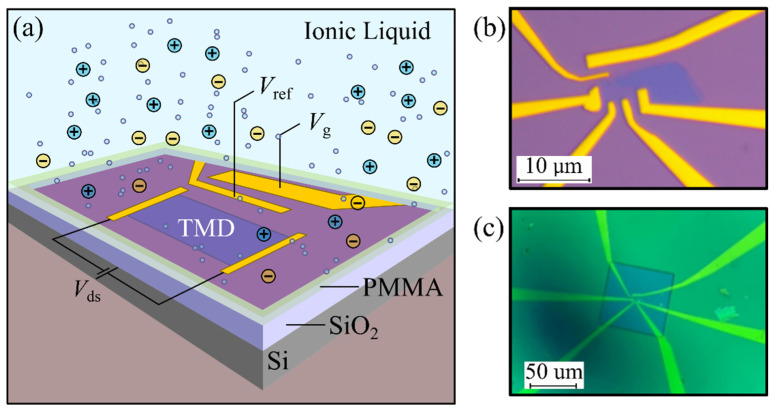
(**a**) Full schematics of an ionic-liquid-gated field-effect transistor (FET), showing the gate and reference electrodes, as well as the electrical circuit used to bias and measure the device. (**b**) Optical microscope image of a bilayer of WSe_2_ contacted in Hall bar configuration (the scale bar is 10 um). (**c**) Optical microscope image of the device’s polymethyl methacrylate (PMMA) windows (the scale bar is 50 um).

Figure 1a schematically shows the geometry of a TMD-based ILG transistor. To illustrate the typical geometry and behavior of this family of transistors, we refer to the device shown in Figure 1b. In our case, the channel is a thin bilayer WSe_2_ crystal, fabricated by standard mechanical exfoliation and ulterior transfer onto a SiO_2_/Si substrate. The metallic electrodes were fabricated by e-beam lithography and evaporation of titanium and gold (5/45 nm). In addition to the four electrodes connected to the WSe_2_ flake, two electrodes were fabricated to act as the gate (*V*_g_) and reference (*V*_ref_) electrodes. As a final step, the whole device was covered with a droplet of ionic liquid (DEME-TFSI), contacting the semiconductor channel, as well as the reference and gate electrodes (see Section S1 in Supplementary Materials for more information on the IL and its deposition). To minimize the exposure of the IL to the gold pads, the whole device was covered with polymethyl methacrylate (PMMA), leaving an exposed rectangular window on top of the semiconductor channel for placing the droplet (see Figure 1c).

### 2.2. Basic Device Operation and Doping Mechanisms

**Figure 2:**
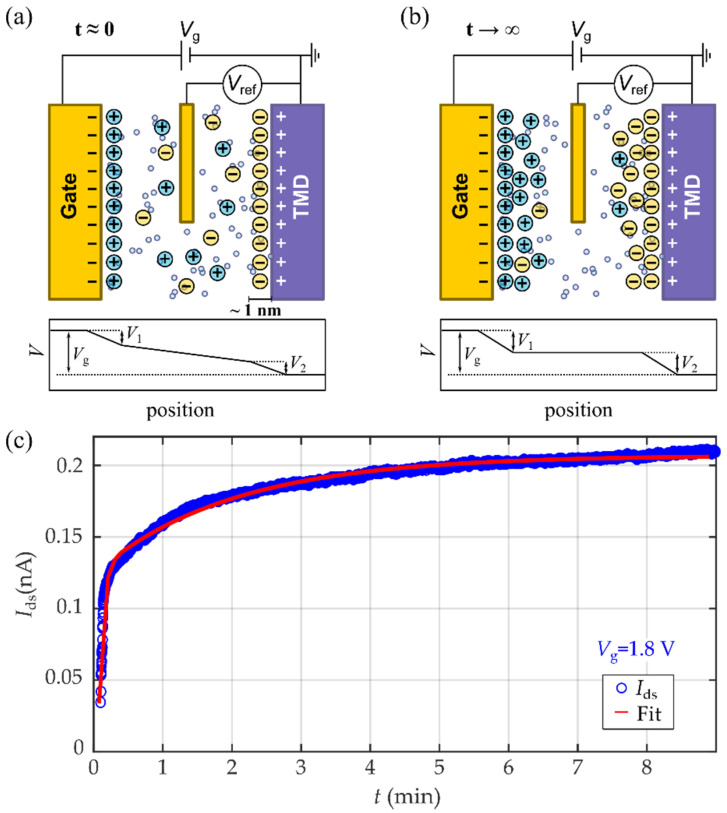
(**a**,**b**) Schematic diagram of the gating mechanism immediately after applying a gate voltage (**a**) and once the electric field inside the ionic liquid is fully screened (**b**). (**c**) Evolution of the drain source current (blue dots), measured immediately after switching *V*_g_ from 0 to 1.8 V. The current progressively increases as the Electrostatic Double Layer (EDL) is formed. The formation process of the EDL can be fitted to the charge process of two plane-parallel capacitors.

The basic operation of the ILG transistor is depicted in Figure 2a,b. When a gate voltage is applied, the finite-sized ions accumulate in consecutive layers close to the TMD channel, forming a nanocapacitor that is typically 1 nm or less. It enhances a large electric field, resulting in a strong gating effect that can be controlled by the application of voltage to the gate electrode.

Figure 2c shows the time evolution of the drain-source current in the few-layer WSe_2_ IL-gated transistor, measured while switching the gate voltage from 0 to 1.8 V. The measured current $I_{\mathrm{ds}}$ can be well-fitted to the equation for the charge process of two plane-parallel capacitors with different characteristic times:

(1) Ids(t)=A+B(1−e−tα1)+C(1−e−tα2)

where τ1,2=1α1,2 are the characteristic times of the formation of the ionic layers that we use as fitting parameters. We obtained the characteristic times of $\tau_{1}=30\;s$ and $\tau_{2}=23\;\min$. These two characteristic times can be associated to the presence of two different charging processes. One is related to the fast formation of the first ion compact shells. The other one is caused by a slower migration and accumulation of ionic species in consecutive layers until the electric field inside the ionic liquid is fully screened [45].

While in early works, the doping effect in IL-gated FETs was attributed solely to the electrostatic screening of the accumulated charges at the interfaces, it is now clear that two main mechanisms govern ionic-liquid gating, depending on the characteristics of both the electrolyte and the material used as a channel [46]: electrostatic doping (described above) and electrochemical doping. For this second mechanism, the migration of ions within the material plays a key role and may induce an irreversible behavior caused by chemical degradation. Electrochemical doping is often the dominant gating mechanism when the IL is used in combination with transition metal oxides. In this case, the doping process also involves the migration of oxygen atoms from the crystallographic unit cell. The oxygen atoms act as dopants, enabling the introduction of charge carriers into the system [47,48,49]. However, in the case of semiconducting TMDs, ionic gating has an almost pure electrostatic effect and does not cause any chemical modification, as long as the applied gate voltage is kept within a suitable range, which results in stable and reversible transistor operation.

### 2.3. The Need for a Reference Electrode

In a conventional metal–oxide–semiconductor field-effect transistor (MOSFET), the applied gate voltage, *V*_g_, uniformly drops across the gate dielectric. However, as depicted in Figure 2a,b and discussed above, in EDL transistors the voltage drop concentrates in the neighboring regions of the gate electrode (*V*_1_) and the channel (*V*_2_). Thus, in equilibrium we have:

(2) $$V_{g}=V_{1}+V_{2},$$

and only a portion of *V*_2_ of the applied voltage, *V*_g_, contributes to gating.

In the hypothetical situation in which *V*_1_ becomes negligible, the applied gate voltage, *V*_g_, drops entirely at the IL/WSe_2_ interface (*V*_2_ = Δ*V*_g_). Experimentally, in ILG measurements, the gate electrode is usually (and intentionally) fabricated to have a large surface area, so the contribution of *V*_1_ can be minimal; however, it cannot be neglected.

In general, *V*_1_ and *V*_2_ do not change linearly with *V*_g_, and, furthermore, they may fluctuate over time and/or present hysteretic behaviors. In consequence, it is necessary to introduce a reference electrode, *V*_ref_, to monitor *V*_2_ situated in contact with the ionic liquid (see Figure 2a,b). For sufficiently long times, once the EDLs are fully formed, *V*_ref_ will be given by:

(3) $$V_{\mathrm{ref}}=V_{g}-V_{1}=V_{2}.$$

Thus, *V*_ref_ provides us with a direct measurement of the voltage drop at the liquid/TMD interface, which is responsible for the gating effect.

### 2.4. Nonmonotonic Behavior in Transfer Characteristics and Estimation of Semiconductor Band Gap

**Figure 3:**
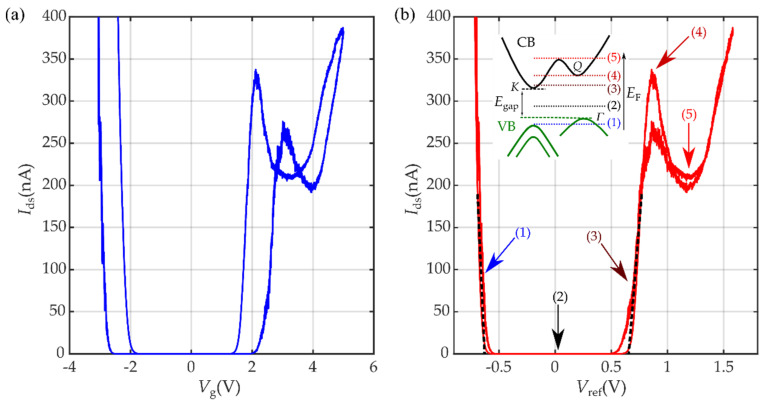
Transfer characteristic of a few-layer WSe_2_ ionic-liquid-gated FET, measured at *V*_ds_ = 0.1 V as a function of (**a**) the gate voltage, *V*_g_, (sweep rate at 1 mV/s) and (**b**) the reference voltage, *V*_ref_. The threshold voltage values for electrons ($V_{\mathrm{th}}^{e}$) and holes ($V_{\mathrm{th}}^{h}$) were determined by linearly extrapolating to zero the *I*_ds_–*V*_ref_ characteristics, as indicated by the black dashed lines. All measurements were taken at 240 K. The inset in Figure 2b depicts a schematic illustration of the conduction and valence band edges of bilayer WSe_2_, showing the *K* and *Q* valleys in the conduction band, as well as the (spin-split) *K* valley and the *Γ* valley in the valence band. The conduction band edge consists of a single line because at the temperature of our experiments (240 K), spin-splitting is smaller than the thermal energy and it can be disregarded. *E*_gap_ indicates the energy distance between the conduction band edges at the *K* and *Γ* valleys. In the valence band in WSe_2_, the high-spin-split *K* valley is lower than the *Γ* valley in terms of binding energy. Colored arrows and numbers depict the position of the Fermi energy at different *V*_ref_ in the transfer curve.

Figure 3 shows the transfer characteristic of a WSe_2_ ILG transistor, measured at 240 K (see Section S2 for measurements at other temperatures). As mentioned in the previous section, when the drain-source current is plotted against the gate voltage, V_G_ (Figure 3a), a large hysteresis appears because of the slow process of ion diffusion in the ionic liquid. However, this hysteresis largely decreases when *I*_ds_ is represented as a function of *V*_ref_ (Figure 3b).

The large shifts in the Fermi energy that can be achieved in ILG transistors allow us to observe ambipolar conduction in the transfer characteristic even while applying moderate gate voltages. A large source-drain current, *I*_ds_, is measured for both high negative and positive *V*_g_. When the Fermi level is in the WSe_2_ band gap (OFF state), the measured current is just 10 pA, indicating there is almost no hopping conductivity because of intragap states or unintentional dopants in the material [41] and confirming the high quality of the WSe_2_ flake.

For positive gate voltages (*V*_g_ > 0), the transfer curve shows a nonmonotonic behavior, also described in the literature using different ionic liquids [50]. This has been found to be related to a nonlinearity that is present in the electron density because of intervalley scattering processes. This intervalley scattering becomes possible when the chemical potential is shifted into a higher energy valley. WSe_2_ bilayers exhibit an indirect band gap between the conduction band minimum at *Γ* and the valence band maximum at *K* in the first Brillouin zone (BZ) [51]. Upon adding electrons, the *K* valley is filled first to above a certain value (denoted by (4) in the inset of Figure 3b), and the *Q* valley also starts to be filled. This inflection point enabled the quantitative determination of the energy difference between the *K* and *Q* valleys of monolayer WSe_2_ in the literature, $E_{Q}-E_{K}=108\;\mathrm{meV}$ [50]. We estimated the energy difference between the *K* and *Q* valleys for bilayer WSe_2_, $E_{Q}-E_{K}$ = 40 meV (see Section S3), to be in agreement with the value obtained in the literature [51]. For negative gate voltages (*V*_g_ < 0), this nonmonotonic behavior is not observed. In this case, the second valley to be depleted of electrons would be the valley centered at *K*. However, the required hole density to reach this second valley is above the values achieved in our measurements.

### 2.5. ILG: A Spectroscopy Technique to Estimate the Semiconductor Band Gap

Currently, determining the band gap of two-dimensional semiconductors is usually undertaken using optical techniques [52,53,54] or by scanning tunneling spectroscopy (STS) [55,56], although complex techniques, such as angle-resolved photoemission spectroscopy (ARPES) [57,58], have also been used. However, these first two commonly used techniques require modeling of the measured data to extract a quantitative value for the gap. In optical techniques, an analysis of excited exciton states is required, this being a hard approach for indirect band gap semiconductors. In the case of the STS, the measured differential conductance must be modeled because the tip acts as a local gate, shifting the energy of the band edge and modifying the probability of electrons tunneling through vacuum.

As recently proved by Morpurgo et al. [53], IL gating can be used as a spectroscopy technique to precisely determine the band gap of a semiconductor from simple transport measurements. Because of the close proximity of the ionic liquid to the semiconductor channel, donor or acceptor impurities are negligible at the interface. Thus, a change in the gate voltage (or more precisely in the reference potential, Δ*V*_ref_) is directly related to a shift in chemical potential, and the difference between $V_{\mathrm{th}}^{e}$ and $V_{\mathrm{th}}^{h}$ is a direct measurement of the semiconductor band gap.

A change in reference voltage induces a change in both the chemical potential, Δμ, and the electrostatic potential, Δφ:

(4) $$e\Delta V_{\mathrm{ref}}\;=\;\Delta \mu +e\Delta \varphi .$$

The electrostatic potential in a parallel-plate capacitor can be defined as:

(5) $$\Delta \varphi =\frac{e\Delta n}{C_{G}},$$

where Δn is the density of accumulated charge carriers at the capacitor plate and $C_{G}$ is the geometric capacitance.

For Fermi energies within the TMD band gap, Δ*n* is small because, ideally, there are no available states to be occupied by charge carriers, and the term Δ*φ* in Equation (4) can be disregarded. In this situation, a shift in gate voltage induces an identical shift in chemical potential:

(6) $$e\Delta V_{\mathrm{ref}}=\Delta \mu .$$

Therefore, the band gap of the semiconductor channel, $E_{\mathrm{gap}}$, can then be determined as:

(7) $$E_{gap}=e\;\left(V_{\mathrm{th}}^{e}-V_{\mathrm{th}}^{h}\right),$$

since $V_{\mathrm{th}}^{e}$ and $V_{\mathrm{th}}^{h}$ correspond to having μ located, respectively, at the conduction and valence band edges.

**Figure 4:**
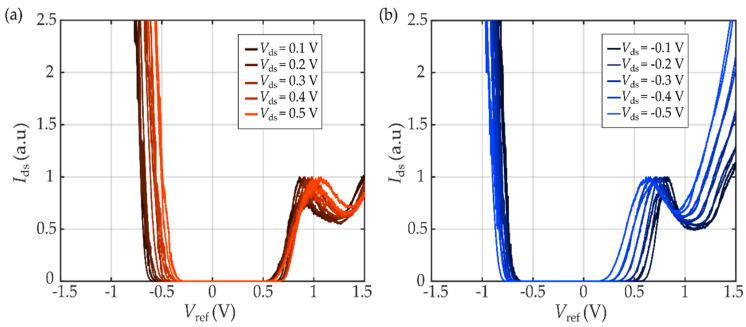
Drain current, *I*_ds_, versus reference voltage, *V*_ref_, at different (**a**) positive and (**b**) negative drain-source voltages, *V*_ds_. The threshold voltage values were determined by linearly extrapolating to zero the *I*_ds_ − *V*_ref_ characteristics and the WSe_2_ energy band gap was estimated by extrapolating *V*_ds_ to zero.

Figure 4 shows the transfer characteristics of the WSe_2_ device measured at different positive (Figure 4a) and negative (Figure 4b) drain-source voltages, *V*_ds_. The threshold voltage values for electrons, $V_{\mathrm{th}}^{e}$, and holes, $V_{\mathrm{th}}^{h},$ were obtained by linearly extrapolating to zero the $I_{\mathrm{ds}}-V_{\mathrm{ref}}$ characteristics, (see black dashed lines in Figure 3b). To perform the extrapolation properly, it is important to identify a sufficiently large range of *V*_ref_ in the linear regime, out of the sub-threshold region, in which *I*_ds_ increases exponentially on *V*_ref_ [59].

The band gap is estimated by extrapolating to $V_{\mathrm{ds}}=0\;V$. We obtain:

$$E_{{\mathrm{WSe}}_{2}}=e\;\left(V_{\mathrm{th}}^{e}-V_{\mathrm{th}}^{h}\right)=1.3\;\mathrm{eV},$$

with an ∼±5% experimental error that originated from the extrapolation procedure. This value agrees with the band gap measured with experimental techniques (1.5−1.6 eV) [51,59,60,61,62], as well as with the value estimated theoretically for bilayer WSe_2_ (1.1 eV) [45]. At high *V*_ds_, linear shifts in the threshold voltage appear. This threshold voltage was previously associated in WS_2_ with uncertainties in the measurements [41] and here we relate it to the position dependence of the reference electrode, its geometry and area indicating the need to measure with low *V*_ds_ because of the strong dependence on the localization of the reference electrode. The leakage current was also measured during the experiment, keeping the values below 0.05 nA (see Section S4 for more information).

## 3. Conclusions

In this work, we described and demonstrated the operation principles of ionic liquid gating in TMD-based transistors. We produced an ambipolar field-effect transistor with bilayer WSe_2_ flake crystals, explaining the importance of the reference voltage, *V*_ref_, for obtaining hysteresis-free transfer characteristics. ILG allowed us to obtain steep subthreshold slopes for both electrons and holes and extremely low OFF-state currents. We obtained evidence of the potential spectroscopic capabilities of ionic-liquid-gated transistors by acquiring the band gap of bilayer WSe_2_ directly from those measurements.

The possibility of quantitatively determining the band gaps and band offsets directly from simple transfer characteristics makes the IL gating a promising new technique, ideal for characterizing 2D semiconductor materials and their heterostructures.

## Data Availability

The data that support the findings of this study are available from the corresponding author upon reasonable request.

## Supplementary Materials

Supplementary material is available online at https://www.mdpi.com/article/10.3390/mi12121576/s1, S1: Deposition of the ionic liquid DEME-TFSI, S2: Transfer curves at different temperatures, Figure S1: Transfer characteristics of the bilayer WSe_2_ ionic liquid-gated transistor, S3: Estimation of the energy splitting between valleys Q-K in bilayer WSe_2_, S4: Measuring the gate leakage current and the linear dependence of *V*_ref_ and *V*_gate_, Figure S2 Gate leakage current, *I*_G_, measured between the gate electrode and the device as function of the reference voltage, *V*_ref_ while sweeping the gate voltage, *V*_g_, at 1 mV/s.
