# Supplementary material for: Ionic-Liquid Gating in Two-Dimensional TMDs: The Operation Principles and Spectroscopic Capabilities
Source: Micromachines (Basel). 2021 Dec 17;12(12):1576. doi: 10.3390/mi12121576 (PMC8704478; doi:10.3390/mi12121576)
Supplement: Supplementary file 1 [file micromachines-12-01576-s001.zip › micromachines-1487839 supplementary.pdf]

Supplementary information

Ionic-liquid gating in two-dimensional TMDs: recent advances and spectroscopic capabilities.

Daniel Vaquero[1], Vito Clericò[1], Juan Salvador-Sánchez[1], Jorge Quereda[1], Enrique Diez[1], Ana M. Perez-Muñoz[1,2]*

1 Nanotechnology Group, USAL–Nanolab, Universidad de Salamanca, E-37008 Salamanca, Spain
2 FIW Consulting S.L, Gabriel Garcia Marquez, 4, las Rozas., E-28232 Madrid, Spain.
* Correspondence: anaperezmu@usal.es

S1. Deposition of the ionic liquid DEME-TFSI

S2. Transfer curves at different temperatures

S3. Estimation of the energy splitting between valleys Q-K in bilayer WSe_2

S4. Measuring the gate leakage current and the linear dependence of V_{ref} and V_{gate}

S1. Deposition of the ionic liquid DEME-TFSI

EDL transistors were fabricated on top of SiO_2/Si substrate where a bilayer of WSe_2 crystal was mechanically exfoliated and trasferred with a standard procedure. The metallic electrodes are defined by e-beam lithography and evaporation of titanium and gold (5/45 nm). Besides the four electrodes connected to the WSe_2 flake, two additional electrodes are fabricated to act as the gate (V_g) and reference (V_{ref}) electrodes. To minimize the exposure off the IL to the contact guides the whole device was covered with polymethyl methacrylate (PMMA) and a rectangular window was open in the center of the channel of the semiconductor for placing the droplet. As a final step, we deposit a drop of the ionic liquid N,N-diethyl-N-(2-methoxyethyl)-Nmethylammonium bis(trifluoromethylsulphonyl)imide (DEME-TFSI) on top of the window, covering the monolayer, the reference and the gate electrode.

The DEME-TFSI shows large capacitance and a wide electrochemical window allowing to achieve extremely large accumulations of charge carriers, up to 5×10^{14} electrons/cm^2 in ILG-based 2D transistors[1]. Owing to the substantial cooling properties of this liquid and its glass transition characteristics the DEME-TFSI is an optimal choice for this experiment.

To prevent the chemical reaction of the surface of the sample with water in the IL, the liquid was baked in vacuum at 80 °C for a period of 24 h. Then, the IL drop was placed on top of the device and the sample was rapidly introduced in the vacuum measurement chamber to avoid long-term air exposure. The system was cooled down and pumped overnight to remove humidity and oxygen and further prevent unwanted chemical reactions and device degradation. The DEME-TFSI freezes below 220 K[2], however, the glass-transition temperature, T_g, occurs at 183K[3]. We carried the experiment at 240K when the IL is in the rubber phase. In this situation ions are still mobile within the liquid but most chemical reactions are suppressed[4]. All the transport measurements were performed in high vacuum, with a pressure of 10^{-6} Pa.

S2. Transfer curves at different temperatures

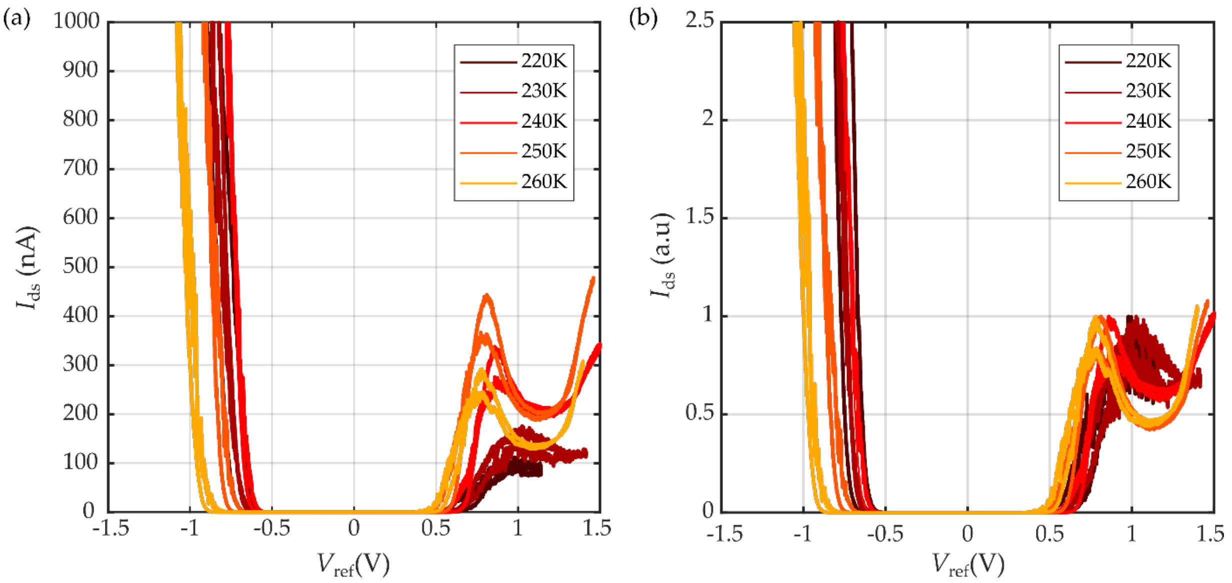

Figure S1. a) Transfer characteristics of the bilayer WSe_2 ionic liquid-gated transistor, measured at V_{ds} = 0.1V as a function of the reference voltage V_{ref} for different temperatures. b) Normalized transfer curves.

Figure S1a shows the transfer characteristics of the 2L-WSe_2 ILG transistor at different temperatures. The range of temperatures has been chosen so the IL (DEME-TFSI) is in the rubber phase, above the glass-transition temperature, T_g = 183 K[3] and below 260K where chemical reactions may influence the measurement. As described in the main text, for positive reference voltages ($V_{ref} > 0$) the transfer curve shows an inflection point related to the nonlinearity present in the electron density. For negative gate voltages ($V_g < 0$) this behaviour is not observed for this range of voltage. Near the inflection point, we can observe that the current increases as we increase the temperature from 220K to 250K. We attribute this effect to the presence of Schottky barriers in the electrical contacts. In order to compare the different transfer curves we normalize I_{ds} to the inflection point (Figure S1b). While the overall shape of the normalized curves is similar for all the temperatures, they shift to more negative voltages as we increase the temperature. We attribute this shift to temperature-dependent alterations in the chemical properties of the liquid. Besides this overall shift, we also observe a slight variation in the position of the inflexion point, relative to V_{th}^{e}, which we associate to a temperature-dependent filling of states at the Q valley.

S3. Estimation of the energy splitting between valleys Q-K in bilayer WSe_2

To estimate the energy splitting between valleys K-Q we use the Fermi-Dirac statistics approximating WSe_2 to a two-dimensional electron gas. The charge density in the device is equal to the density of occupied states in the conduction band, $\Omega(E_F)$.

$$\Omega(E_F) = n = \int_{-\infty}^{\infty} g_{2D}(E)\, f(E,\mu)\, dE \tag{S1}$$

where, $f(E,\mu)$ is the Fermi-Dirac distribution at 240 K and $g_{2D}(E)$ is the density of states of a two-dimensional free electron Fermi gas, given by

$$g_{2D}(E) = \begin{cases} \frac{m_e^*}{2\pi\hbar} g_s g_v & if\ E > E_{CB} \\ 0 & if\ E < E_{CB} \end{cases} \tag{S2}$$

where m_e^* is the effective mass of the electron in the conduction band, $g_s = 2$ is the spin degeneracy and $g_v = 2$ is the valley degeneracy. We select the bottom of the conduction band as the origin of energies, $E_{CB} = 0$.

Then, the equation S1 yields,

$$n = \frac{2m_e^*}{\pi\hbar} \int_0^{\infty} f(E)\, dE \tag{S3}$$

In order to bring the fermi energy from the bottom of the K valley, $E_F = E_{CB} \equiv 0$, to the bottom of the Q valley, $E_F = E_Q$, one needs to increase the charge density by

$$\Delta n = \frac{2m_e^*}{\pi\hbar} \int_0^{E_Q} f(E)\, dE. \tag{S4}$$

We can now estimate Δn from the measured transfer curves as

$$\Delta n = \frac{C_{IL}}{e} \Delta V_{ref} \tag{S5}$$

Where e is the electron charge, ΔV_{ref} is the variation of the reference voltage between the two valleys (*i.e.* the difference between $V^e{}_{th}$ and the inflection point, labelled as (4) in Figure 3b of the main text) and C_{IL} is the capacitance of the ionic liquid. In our case we get $\Delta V_{ref} = 0.23\ V$ and we use a value of the capacitance of $C_{IL} = 10\ \mu F/cm^2$ similar to previous literature [5–7].

Finally comparing the right-hand terms of equations S4 and S5 we obtain,

$$\int_0^{E_Q} f(E)\, dE = \frac{C_{IL}}{e} \Delta V_{ref} \frac{\pi\hbar}{2m_e^*} \tag{S6}$$

This equation is then solved numerically to obtain $E_Q - E_K = 40$ meV, in good agreement with values previously reported in the literature for bilayer WSe_2 [8].

S4. Measuring the gate leakage current and the linear dependence of V_{ref} and V_{gate}

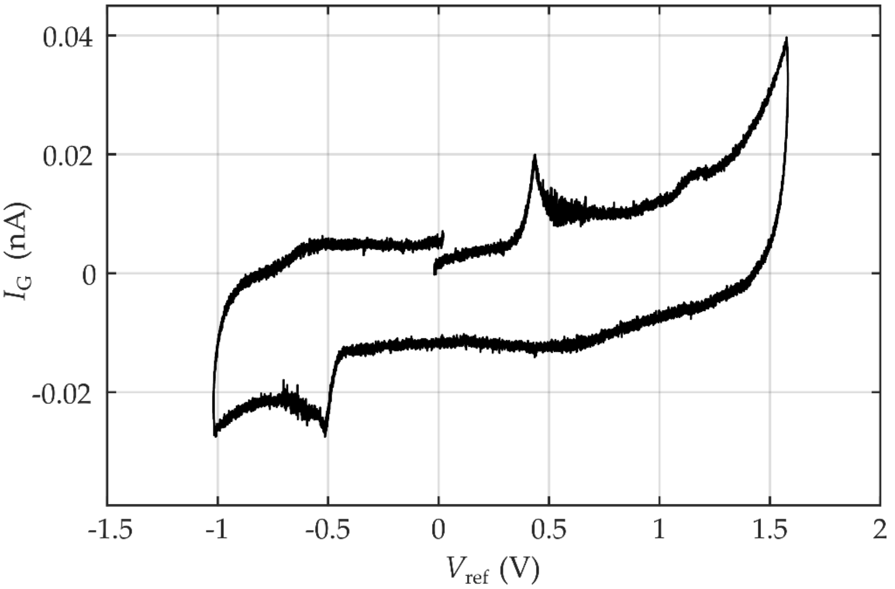

Figure S2. Gate leakage current, I_G, measured between the gate electrode and the device as function of the reference voltage, V_{ref} while sweeping the gate voltage, V_g, at 1mV/s.

A key aspect to be considered experimentally is that the electrolyte has to be equipotential (without current flowing through it) to guarantee the analysis done in the main text, $e\Delta V_{ref} = \Delta\mu$ (eq 6), that allows us to precisely determine the bandgap. Figure S2 shows a negligibly small gate leakage current, I_G, measured when the device was operating at V_{ds}= 0.1V. I_G was measured between the gate electrode and the device while sweeping V_g at 1 mV/s to avoid an increase in the I_G and the appearance of chemical reactions. This current is mainly caused by charging process of the capacitors formed at electrolyte/gate and electrolyte/semiconductor interfaces. The peaks in I_G at approximately V_{ref}~ −0.5 V and V_{ref} ~ +0.5 V correspond to a capacitance change associated with, respectively, the accumulation of holes and electrons at the surface of WSe_2[9]. The distance between the peaks is ~1 eV, close to the value of E_{GAP} = 1.3 eV extracted from the FET characteristics.

References

(1) Bisri, S. Z.; Shimizu, S.; Nakano, M.; Iwasa, Y. Endeavor of Iontronics : From Fundamentals to Applications of Ion-Controlled Electronics. **2017**, *1607054*, 1–48. https://doi.org/10.1002/adma.201607054.

(2) Yuan, H.; Shimotani, H.; Tsukazaki, A.; Ohtomo, A.; Kawasaki, M.; Iwasa, Y. High-Density Carrier Accumulation in ZnO Field-Effect Transistors Gated by Electric Double Layers of Ionic Liquids. *Adv. Funct. Mater.* **2009**, *19*, 1046–1053. https://doi.org/10.1002/adfm.200801633.

(3) Sato, T.; Masuda, G.; Takagi, K. Electrochemical Properties of Novel Ionic Liquids for Electric Double Layer Capacitor Applications. *Electrochim. Acta* **2004**, *49* (21), 3603–3611. https://doi.org/10.1016/j.electacta.2004.03.030.

(4) Awaga, T. F. & K. Electric-Double-Layer Field-Effect Transistors with Ionic Liquids. *Phys. Chem. Chem.Phys* **2013**, No. 15, 8983–9006. https://doi.org/10.1039/C3CP50755F.

(5) Matsumoto, M.; Shimizu, S.; Sotoike, R.; Watanabe, M.; Iwasa, Y.; Itoh, Y.; Aida, T. Exceptionally High Electric Double Layer Capacitances of Oligomeric Ionic Liquids. *J. Am. Chem. Soc.* **2017**, *139* (45), 16072–16075. https://doi.org/10.1021/jacs.7b09156.

(6) Zhang, H.; Berthod, C.; Berger, H.; Giamarchi, T.; Morpurgo, A. F. Band Filling and Cross Quantum Capacitance in Ion-Gated Semiconducting Transition Metal Dichalcogenide Monolayers. *Nano Lett.* **2019**, *19* (12), 8836–8845. https://doi.org/10.1021/acs.nanolett.9b03667.

(7) Wang, F.; Stepanov, P.; Gray, M.; Lau, C. N.; Itkis, M. E.; Haddon, R. C. Ionic Liquid Gating of Suspended MoS2 Field Effect Transistor Devices. *Nano Lett.* **2015**, *15* (8), 5284–5288. https://doi.org/10.1021/acs.nanolett.5b01610.

(8) Zhao, W.; Ribeiro, R. M.; Toh, M.; Carvalho, A.; Kloc, C.; Castro Neto, A. H.; Eda, G. Origin of Indirect Optical Transitions in Few-Layer MoS2, WS2, and WSe2. *Nano Lett.* **2013**, *13* (11), 5627–5634. https://doi.org/10.1021/nl403270k.

(9) Braga, D.; Gutiérrez Lezama, I.; Berger, H.; Morpurgo, A. F. Quantitative Determination of the Band Gap of WS_2 with Ambipolar Ionic Liquid-Gated Transistors. *Nano Lett.* **2012**, *12*, 5218–5223. https://doi.org/10.1021/nl302389d.
